# Evaluation of an acceleration-based assistive strategy to control a back-support exoskeleton for manual material handling

**DOI:** 10.1017/wtc.2020.8

**Published:** 2021-01-11

**Authors:** Maria Lazzaroni, Ali Tabasi, Stefano Toxiri, Darwin G. Caldwell, Elena De Momi, Wietse van Dijk, Michiel P. de Looze, Idsart Kingma, Jaap H. van Dieën, Jesús Ortiz

**Affiliations:** 1 Department of Advanced Robotics, Istituto Italiano di Tecnologia, Genova, Italy.; 2 Department of Electronics, Information and Bioengineering, Politecnico di Milano, Milano, Italy.; 3 Department of Human Movement Sciences, Faculty of Behavioural and Movement Sciences, Vrije Universiteit Amsterdam, Amsterdam, The Netherlands.; 4 TNO, Leiden, The Netherlands.

**Keywords:** occupational back-support exoskeleton, control strategy for active exoskeletons, lifting, manual material handling, musculoskeletal disorders

## Abstract

To reduce the incidence of occupational musculoskeletal disorders, back-support exoskeletons are being introduced to assist manual material handling activities. Using a device of this type, this study investigates the effects of a new control strategy that uses the angular acceleration of the user’s trunk to assist during lifting tasks. To validate this new strategy, its effectiveness was experimentally evaluated relative to the condition without the exoskeleton as well as against existing strategies for comparison. Using the exoskeleton during lifting tasks reduced the peak compression force on the L5S1 disc by up to 16%, with all the control strategies. Substantial differences between the control strategies in the reductions of compression force, lumbar moment and back muscle activation were not observed. However, the new control strategy reduced the movement speed less with respect to the existing strategies. Thanks to improved timing in the assistance in relation to the typical dynamics of the target task, the hindrance to typical movements appeared reduced, thereby promoting intuitiveness and comfort.

## Introduction

Musculoskeletal disorders (MSDs) are the most frequent occupational disease in many industrialized countries (Punnett and Wegman, 2004; Bevan, 2012; Parent-Thirion et al., 2016), with significant socioeconomic impact on individuals and health care systems (Davies et al., 2003; Woolf and Pfleger, 2003; Hoy, 2014). Common to many industrial sectors, manual material handling (MMH) tasks increase the risk of developing MSDs associated with the back (Zurada, 2012).

### Biomechanics of Back-Related MSDs

Back-related MSDs are associated with mechanical overloading and compression on the spine (Kumar, 2001; Coenen et al., 2014). During MMH, spinal muscles and passive tissues must generate large extensor moments, resulting in large compression forces on lumbar discs (Dolan et al., 1994).

Due to the difficulty in quantifying the reduction of MSDs, an easier approach involves the assessment of the risk factors identified as increasing MSDs incidence during MMH. The extensor moment about the lumbar joint indicates the response of the musculoskeletal system to the external load applied and largely determines spine compression (Van Dieën and Kingma, 2005). Peak and cumulative extensor moments have been identified among the factors that increase the risk of developing MSDs during lifting tasks (Marras et al., 1995; Norman et al., 1998)).

In the same studies, trunk movement velocity was identified as another of the factors that increase MSDs risk. Moreover, peak L5S1 moment increases with the increasing lifting speed (Bush-Joseph et al., 1988; Greenland et al., 2013), while the cumulative moment is higher at lower speeds (Greenland et al., 2013) because of the longer total lift duration. In Granata and Marras (1995), a significant increase of the spinal compression with the lifting speed was observed and especially for the lowering phase (Davis et al., 1998).

The trunk inclination angle directly affects the extensor moment. Indeed, during trunk bending, compression force on the L5S1 lumbar disc increases with increasing inclination of the trunk (Andersson et al., 1976; Toxiri et al., 2018) because spinal muscles and passive tissues must balance the increasing moment due to gravity.

For lumbar and thoracic erector spinae muscles, a relationship between their activity and lumbar extensor moment was observed by Potvin et al. (1996) and Dolan and Adams (1993). Therefore, recording the electromyography (EMG) signal is currently the most common measure to monitor lumbar load during load handling tasks, especially over long durations (Potvin et al., 1996). Moreover, the contribution due to erector spinae muscles on lumbar extensor moment is dominant, although the passive contribution to the extensor moment (involving the intervertebral disc and ligaments, the iliolumbar ligaments, the lumbo-dorsal fascia and collagenous tissue within the muscles) also generates compression on the spine (Dolan et al., 1994).

### Back-Support Exoskeletons

To reduce the incidence of back-related occupational MSDs, back-support exoskeletons have been introduced (de Looze et al., 2016; Toxiri et al., 2019) to assist MMH, mainly focused on load lifting and lowering. The use of passive exoskeletons (Abdoli-e and Stevenson, 2008; Bosch et al., 2016; de Looze et al., 2016) and active exoskeletons (Muramatsu et al., 2013; de Looze et al., 2016; Chen et al., 2018; Huysamen et al., 2018; Ko et al., 2018; Koopman et al., 2019c) has been associated with reductions of spinal muscle activity (up to 40%). Correspondingly, reductions (up to 20%) in the compression of the spine have been estimated by Koopman et al. (2019a), Koopman et al. (2019c), and Frost et al. (2009).

For passive exoskeletons, the amount of assistance is set as part of the mechanical design, and it can only be changed via manual adjustments (e.g., via set screws). Moreover, they only store and release energy provided by the wearer. By contrast, active exoskeletons can inject external energy, modulating the assistance provided online by means of appropriate control strategies. This aspect could potentially enhance the effectiveness and versatility of active exoskeletons compared to passive ones.

Different combinations of sensors have been used to detect the user’s movement intention and accordingly define control strategies that command the actuators to assist the user following the task requirements. The most prevalent methods to control back-support exoskeletons for load handling are based on mechanically intrinsic signals and muscle signals (Koller et al., 2018). Exoskeletons controlled by mechanically intrinsic signals use sensors embedded in the device structure (e.g., inertial measurement units (IMUs), encoders or force/torque sensors) to capture valuable information about the user’s movement, such as joint angles, segment inclinations, velocity, and acceleration. To assist lifting and lowering tasks, the user’s trunk inclination is widely employed to implement gravity compensation (Robo-Mate, Toxiri et al., 2018; Hyundai Waist Exoskeleton (H-WEX), Ko et al., 2018; and HAL, Hara and Sankai, 2010). Another approach is to first detect the beginning of lifting and then control the actuators based on hip and thigh angles (as implemented for Active Pelvis Orthosis (APO), Chen et al., 2018).

Exoskeletons controlled with EMG-based strategies actuate the device according to the user’s muscle activity, anticipating the user’s movement, as EMG signals precede force generation. For back-support exoskeletons, a straightforward method might be a control strategy based on spinal muscle activity, as these muscles are directly responsible for most of the lumbar compression (e.g., HAL exoskeleton, Hara and Sankai, 2010). However, access to spinal muscles using surface EMG (sEMG) might be obstructed by the exoskeleton’s own structure, potentially inhibiting adoption. A different approach is to use a group of muscles that contain valuable information for assisting the task, but it is more accessible for measuring. For this purpose, an sEMG Myo armband was used to measure the forearm muscle activity and accordingly modulate the assistance (Robo-Mate exoskeleton, Toxiri et al., 2018), since the activation of these muscles increases with the weight of the external load being handled.

To effectively assist tasks execution, and thus promote the adoption of back-support exoskeletons in real working scenarios, an open challenge is the selection of the control strategy that best assists the user in executing a specific task. Considering the use in industrial settings, the main factors to select a control strategy are its practical functionality (considering the user’s freedom of movement and wearability and ease of use) and its effectiveness in reducing MSDs risk factors.

### Objective of this Study

The aim of this study is to investigate the effects of different control strategies for a back-support exoskeleton used to assist with lifting and lowering tasks. With respect to previous studies performed on an earlier version of the same device (Toxiri et al., 2018; Koopman et al., 2019c), a new control strategy making use of the angular acceleration of the user’s trunk was introduced in Lazzaroni et al. (2019). The main advantages in terms of assistive torque provided to the users would emerge during the transition phases (i.e., beginning and end of lowering and lifting), thereby providing an appropriate adaptation to their movement dynamics. To validate this new strategy, its effectiveness was experimentally evaluated in terms of spine kinematics, muscle activation, lumbar extensor moment, and compression reductions and variations in the task execution relative to the condition without the exoskeleton. Additionally, to study its effects in greater detail, the acceleration-based strategy was tested against existing strategies for comparison (Toxiri et al., 2018; Koopman et al., 2019c).

## Methods

### Back-Support Exoskeleton

**Figure 1.**
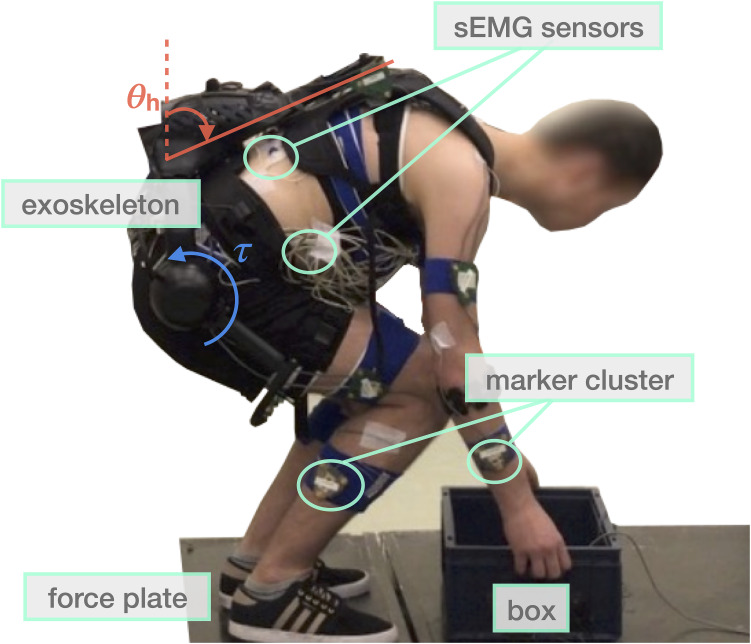
The experimental setup displaying the two force plates, the marker clusters, the electromyography sensors, and XoTrunk exoskeleton. The inclination angle of the trunk 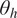 as measured by the onboard inertial measurement units is defined as equal to 0 when the user is standing upright. The actuators generate torques 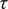 in the sagittal plane between the user’s torso and thighs.

XoTrunk is an active back-support exoskeleton designed to assist workers while performing MMH tasks. The aim is to reduce lumbar overload by generating part of the extensor moment normally generated by the spinal muscles. The prototype is shown in Figure 1. It is an evolution of the Robo-Mate active exoskeleton used in previous studies (Toxiri et al., 2015) and was developed at XoLab, Istituto Italiano di Tecnologia, within a research collaboration with INAIL (Italian Workers’ Compensation Authority).

The device consists of an articulated aluminum frame connected to the user’s body with backpack-like shoulder and waist straps and custom thigh support structures. Two electric actuators are located approximately at hip height, one on each side, and are responsible for generating the assistive torques between the torso and corresponding thigh links in the sagittal plane. The device weighs approximately 6 kg.

**Figure 2.**
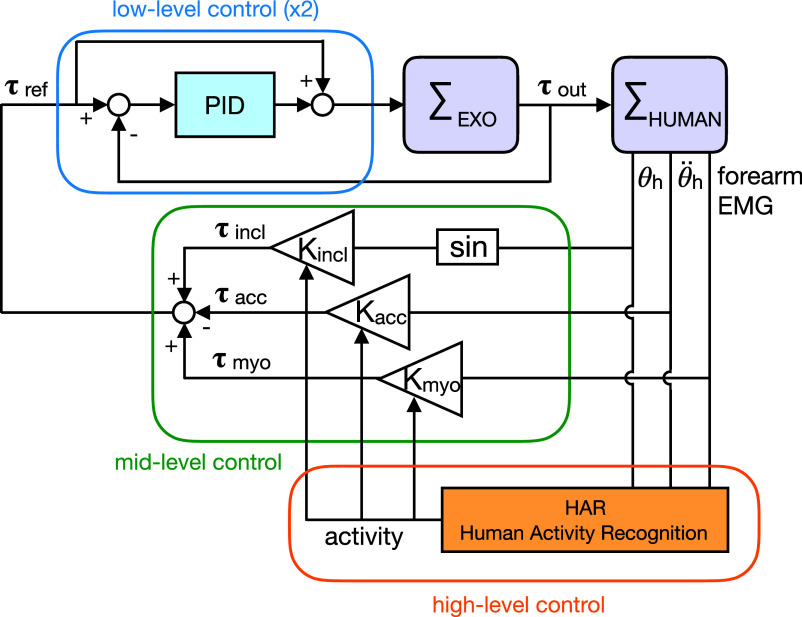
Block diagram representation of the three-levels control system. The high-level control distinguishes the *activity.* The mid-level control modulates the reference torque 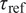 required for the specific activity identified. The low-level control regulates the actuators output 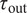 . The exoskeleton 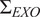 (as an admittance) and the human 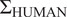 (as an impedance) are mechanically interconnected.

The control scheme is structured on three levels (Figure 2), as proposed in Tucker et al. (2015). The concept that drives the control is based on exploiting the versatility of an active device, by adapting the exoskeleton behavior to the various tasks performed by users. Indeed, different studies (Baltrusch et al., 2018; Näf et al., 2018) have highlighted that passive exoskeletons could interfere with, or restrict, users’ movements during the execution of tasks for which they were not originally designed (e.g., walking, sitting, rotating, squatting, and wide standing).

The idea of the three-levels control (Figure 2) is to assist users only when performing tasks that need assistance and, thus, to overcome the problem of imposing undesirable constraints or obstruction on the execution of tasks that do not require assistance. For this purpose, the high-level control classifies online and in realtime, the different activities the users are performing, automatically distinguishing between walking, standing, and bending (Poliero et al., 2019). The mid-level (i.e., the control strategy) modulates the assistance 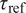 during the execution of the task, adjusting to conditions that determine the need for assistance. Control strategies make use of different signals coming from the users (e.g., trunk angle and trunk acceleration) to estimate their movement intention and accordingly anticipate and adapt the assistance provided by the exoskeleton. Thanks to this control structure, it is possible to select the most convenient control strategy for each recognized task and correspondingly set the control parameters. Finally, the low-level control regulates the output torque of the two actuators 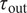 with a closed-loop torque controller, tracking the reference signal 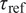 generated by the control strategy.

### Control Strategies for Lowering and Lifting Tasks

Different control strategies have been implemented on the XoTrunk exoskeleton to assist users during lowering and lifting tasks. These strategies consider the factors (one or a combination of them) identified as affecting the lumbar compression: the user’s trunk inclination, the weight of the object lifted, and the user’s trunk angular acceleration.

The *inclination* control strategy (Toxiri et al., 2018) provides assistance proportional to the trunk inclination. The idea is to assist users to partially compensate for the effect of muscle force on spine compression. When the user is bending forward, the spinal muscles and the passive tissues must counteract the moment generated by gravitational forces because of the exoskeleton, the user’s upper body, and the external load masses. The assistive torque is defined proportional to the sine of the user’s trunk angle 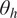 in the sagittal plane ( 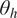 = 0 when the user is standing upright), acquired with an IMU embedded on the exoskeleton’s rigid back structure:
(1)

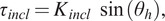

where 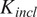 is the control gain that may be adjusted for each user and task to suit individual preferences (e.g., comfort and perceived pressure) and task conditions.

The *hybrid* control strategy (Toxiri et al., 2018) targets two of the factors identified as affecting the lumbar compression: the trunk inclination and the weight of the object lifted. To the *inclination* assistive torque, another torque is added, which is proportional to the EMG of the forearm muscles. This is used as, during grasping and holding, the forearm muscle activity increases with the weight of the object lifted. The activity of these muscles is recorded via a commercially available device based on sEMG: the Myo gesture control armband10 (Thalmic Labs, Inc., Kitchener ON, Canada). The contribution of the two torques is regulated by the corresponding gain 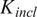 and 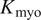 (adjustable for each user and task):



(2)

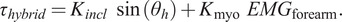



The *dynamic* control strategy (Lazzaroni et al., 2019) adapts the assistance to the dynamics of the movement. This control strategy sets the assistance level to be proportional to both the inclination and the angular acceleration of the user’s trunk. An xSens MTw IMU (Xsens Technology) is attached to the user’s trunk (approximately at the sternum) to measure the trunk angular velocity 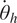 . The trunk angular acceleration 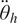 [ 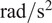 ] is then obtained by differentiating and filtering the angular velocity in the sagittal plane (low-pass filter with a cut-off frequency of 1 Hz). By summing up an inclination-based and an acceleration-based torque, it is possible to assist the user according to his/her statics and dynamics. The static (i.e., 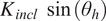 ) and the dynamic (i.e., 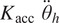 ) contribution can be scaled differently, adjusting the corresponding control gains 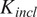 and 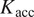 :



(3)

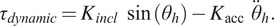



Loosely speaking, the acceleration-based torque may be seen as compensating for part of the inertia of the user’s upper body, while the inclination-based torque compensates for its mass.

**Figure 3.**
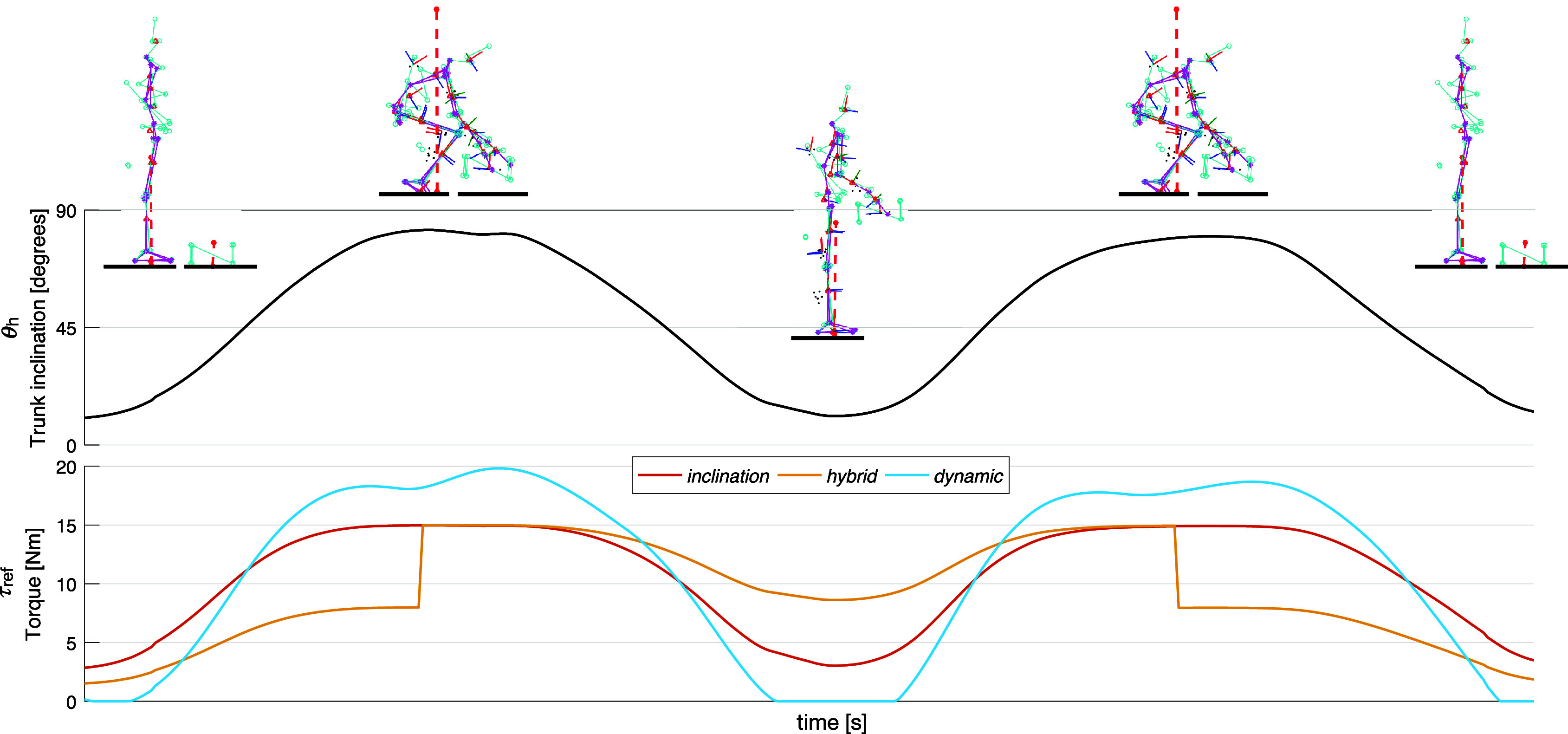
Torque references generated by the *inclination* (red), *hybrid* (yellow), and *dynamic* (blue) control strategy during idealized lifting and lowering tasks. The same reference torque is used for the two actuators, so the total torque applied at the lumbar joint is double. The trunk inclination and the different phases of the task are displayed at the top.

Figure 3 conceptually illustrates the torques defined by the three control strategies during the different phases. With respect to the *inclination* control strategy, the main advantage of the *hybrid* strategy emerges when the user grasps and holds the box. Greater assistance is provided at the beginning of lifting when the user grasps the box and starts to lift it, that is, when the muscles have to generate the greatest extensor moment (Koopman et al., 2018). Furthermore, a certain assistance is provided also in the upright posture, if the user is holding the box. On the other hand, the main advantage of the *dynamic* strategy emerges during the transition phases of the movement (i.e., beginning and end of lifting and lowering). Indeed, using the user’s trunk angular acceleration signal, it is possible to follow the intention of the user, responding to changes in velocity, which correspond to the will to start or to slow down the movement in a certain direction. In particular, the *dynamic* strategy provides a lower torque at the beginning of lowering because a higher assistance may be perceived as hindering the user in bending over. Furthermore, a greater assistance is provided at the beginning of lifting, when the muscles have to generate the greatest extensor moment (Koopman et al., 2018).

**Figure 4.**
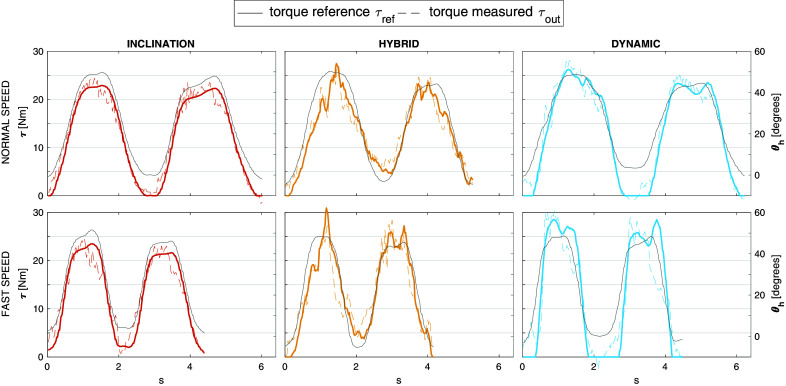
Actual torques applied by the exoskeleton (dashed lines), as measured by torque sensors, compared with the reference torques commanded by control strategies (solid lines) for normal and fast speed. Trunk inclination is displayed in grey.

For illustration purposes, in Figure 4, the actual torque applied by the exoskeleton is compared with the reference torque commanded by the control system (for one subject). In this regard, the improvement of the new prototype with respect to the device we used in our previous study (Koopman et al., 2019c) is visible in terms of torque tracking.

## Evaluation

We devised an experimental protocol to explore the effects of the exoskeleton assistance and more specifically of the *dynamic* strategy compared to previously implemented strategies. Their effectiveness in supporting the user during lifting and lowering tasks was evaluated with particular consideration of the risk factors for the development of MSDs: lumbar extensor moment, spine kinematics (trunk flexion velocity and inclination angle), muscular activation, and lumbar compression force.

The experiment tested two hypotheses. (a) We hypothesized that the physical support provided by the exoskeleton reduces the activation of the spinal muscles during the target task, resulting in reduced compression force and lumbar extension moment (as it would reduce the part of compression specifically attributed to the activation of spinal muscles). (b) The *dynamic* control strategy is compared with the existing strategies. The new control strategy is expected to provide more appropriate support during lowering and lifting tasks, reducing hindrance, and improving the timing of the assistance in relation to the typical dynamics of the movement. This will have a positive impact on increasing the movement speed and reducing the peak compression force. As an additional secondary aspect of interest, we expected changes in the execution of the task when wearing the exoskeleton (e.g., reduced movement speed or trunk inclination) that could result in further advantages or disadvantages for preventing the risk of injuries.

### Experimental Procedures

Nine male healthy subjects (age: 27.3 ± 2.7 years, weight: 73.8 ± 7.6 kg, height: 1.82 ± .09 m) with no history of low back pain participated in the experiments, approved by the local ethics committee. Participants were instructed to perform a complete task defined as the lifting and the lowering of a box (as illustrated in Figure 3). No instructions on the techniques (i.e., stoop or squat) for the lowering and lifting movement were given. The task was executed in four different assistance modes:

- without the exoskeleton: *no-exo*;
- with the exoskeleton: *inclination* strategy (Eq. (1)) with 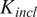 = 15;
- with the exoskeleton: *hybrid* strategy (Eq. (2)) with 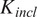 = 10 and 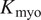 = 10;
- with the exoskeleton: *dynamic* strategy (Eq. (3)) with 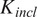 = 15 and 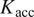 = 1.

To allow comparison between subjects, we empirically selected the values for the gains 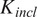 , 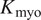 _,_ and 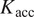 , instead of adjusting them to each subject’s individual preference and body characteristics. Additionally, two different execution speeds (normal and fast) and two different box weights (7.5 and 15 kg) were used. The speed was not strictly imposed. For the normal speed, participants were asked to move at a natural self-selected pace. For the fast speed, participants were asked to perform the task at a substantially faster pace compared to the normal one. For each condition (i.e., each combination of the three independent variables: assistance mode, speed, and box weight), the task was repeated twice. The order of the assistance mode, task execution speed, and box weight were randomized.

### Instrumentation and Data Processing

Subject kinematics were measured with an optoelectronic 3D motion capture system (Certus, Optotrak, Norton Digital, Inc.), with a sampling frequency of 50 Hz. LED cluster markers were attached to lower legs (with feet), upper legs, pelvis, trunk, upper arms, and forearms (with hands). To track the position and orientation of the subject’s body segments and construct the linked segments model, the markers were related to the anatomical landmarks, acquired using pointer measurements (Cappozzo et al., 1995). Ground reaction forces (GRFs) were recorded at a sampling frequency of 200 Hz with two custom-made force plates (1.0 × 1.0 m). Optotrak and force plate data were low-pass filtered with a zero-phase forward-backward second-order Butterworth digital IIR filter, with a cut-off frequency of 10 Hz.

EMG of six spinal and abdominal muscles (rectus abdominis (RA), external oblique (EO), internal oblique (IO), longissimus thoracis (LT), iliocostalis lumborum (IL), longissimus lumborum (LL)) were recorded bilaterally (right and left) with 12 pairs of surface EMG electrodes, placed following SENIAM guidelines (Stegeman and Hermens, 2007). EMG data were amplified (Porti-17TM, TMSi, Enschede, The Netherlands), band-pass filtered (10–400 Hz) with a zero-phase forward–backward second-order Butterworth digital IIR filter, filtered to remove the electrical noise at 50 Hz (forward–backward second-order Butterworth band-stop filter) and the ECG signal (high-pass filter to remove heart rate artifact [Drake and Callaghan, 2006]). The signals, then, were rectified and low-pass filtered with a cut-off frequency of 2.5 Hz (forward second-order Butterworth digital IIR filter) (Potvin et al., 1996) to obtain the envelope. To compare muscle activity levels and activation patterns between muscles, tasks, and individuals, EMG signals were normalized to the maximal voluntary contraction (MVC) (Halaki and Ginn, 2012). To obtain spinal and abdominal muscles’ maximum activity, subjects performed six maximum exertions, repeated twice (McGill, 1991; Vera-Garcia et al., 2010). For spinal muscles MVC acquisition, subjects were strapped in a prone position, with the torso hanging over the edge of the test bench, and asked to extend the trunk upward and to twist right and left against manual resistance applied by the experimenter. To measure the MVC of abdominal muscles, subjects laid in a supine position and attempted to flex the trunk upward and to twist right and left against manual resistance. The torques applied by the exoskeleton were measured using embedded strain gauge-based torque sensors.

### Data Analysis and Statistics

The effects of the exoskeleton and the different control strategies on assisting lowering and lifting were investigated comparing variables that are crucial for defining the risk of low back disorders: trunk inclination angle and velocity, spinal muscle activity, lumbar extensor moment, and compression forces.

With GRFs and the kinematics of the lower body segments, the total L5S1 extensor moment generated by the subject plus the exoskeleton was computed solving the inverse dynamics of a whole-body 3D-linked segment model (Kingma et al., 1996), using bottom-up analysis (Hof, 1992). Then, the net L5S1 extensor moment generated by the subject was calculated by subtracting the torque provided by the exoskeleton from the total L5S1 extensor moment. The lumbar compression force was calculated with an EMG-driven model (Van Dieën and Kingma, 2005). The moments generated by the muscles were estimated using EMG signals and the L5S1 moment obtained solving the inverse dynamics and an optimization procedure. The moments generated by the muscles were distinguished between the moment generated by the abdominal muscles, and the moment generated by the back muscles, and the passive moment generated by the muscles and passive tissues, from which the compression force on the L5S1 joint is computed (Van Dieën and Kingma, 2005).

Peak values of the total L5S1 extensor moment (M 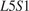 _total), the net L5S1 extensor moment generated by subject (M 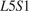 _subj), averaged IL and LL activity, average RA and EO activity, compression force on L5S1, trunk inclination angle, and trunk angular velocity were computed for statistical analysis for all the subjects and conditions. The statistical significance was tested using three-way ANOVA for testing the main effects of the assistance mode, the execution speed, and the box weight, plus their interactions. For variables with significant main effect of the assistance mode (*p*-value 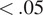 ), a Bonferroni posthoc test was conducted for each combination of speed and box weight separately, to compare the effects of the different assistance modes. Data analysis was performed in Matlab 2018a (The Mathworks, Natick, USA).

## Results

The averages of the peak values across all subjects for the total L5S1 moment, the net L5S1 moment generated by the subject, the averaged IL and LL activity, the compression force on L5S1, the trunk inclination angle, and the trunk angular velocity were tested using three-way ANOVA. Results of the ANOVA tests are shown in Table 1. For all variables, a main effect of the assistance mode was found (*p*-value 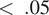 ). A main effect of the execution speed was found for all the variables (the L5S1 moment, the spinal muscle activity, and thus the compression force increases for faster speed). Moreover, a main effect of box weight was found for all the variables, except for the trunk inclination angle (the L5S1 moment, the spinal muscle activity, and thus the compression force increases with the increasing of the box weight).

**Table 1.** *p*-Values of three-way ANOVA tests with factors: assistance mode (*no-exo*, *inclination*, *hybrid,* and *dynamic*), execution speed (normal and fast), and box weight (7.5 and 15 kg) and their interactions

	Main effect	Main effect	Main effect	Interaction	Interaction	Interaction
	Assistance	Speed	Weight	Assistance*speed	Assistance*weight	Speed*weight
M 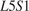 _total	**<.001** ^a^	**<.001**	**.002**	.447	.062	.059
M 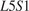 _subj	**<.001** ^a^	**<.001**	**.011**	.676	.139	.2436
EMG IL + LL	**<.001** ^a^	**<.001**	**.004**	.667	.159	.424
Compression	**<.001** ^a^	**<.001**	**<.001**	**.024**	**.012**	.475
Trunk inclination	**.002** ^a^	**.010**	.681	.648	.535	.953
Trunk velocity	**<.001** ^a^	**<.001**	**.041**	.110	**.013**	.110

**Figure 5.**
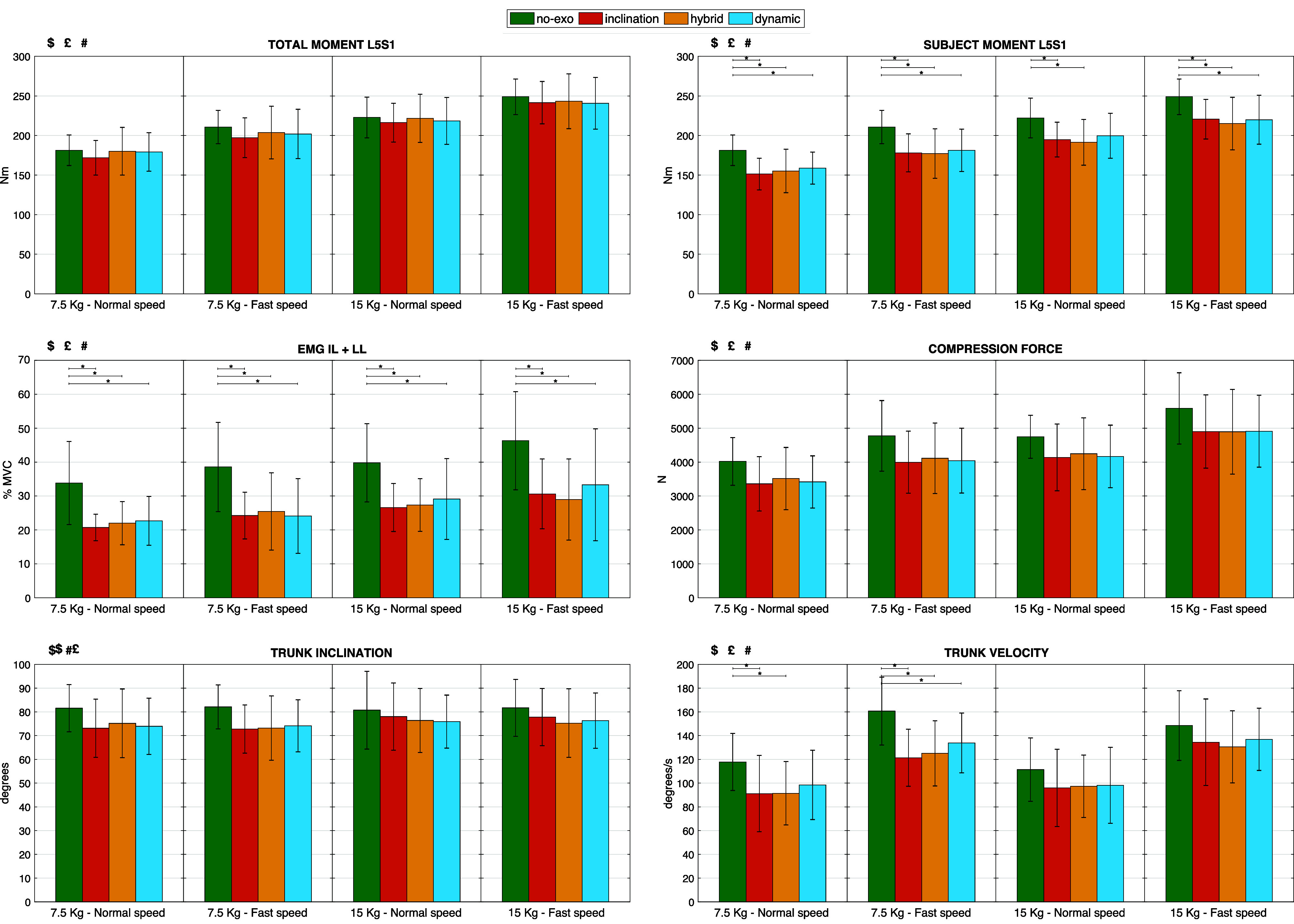
Peaks average and standard deviation across all subjects for variables with a main effect of the assistance mode (indicated with $): total L5S1 moment, net L5S1 moment generated by the subject, averaged iliocostalis lumborum and longissimus lumborum activity, compression force on L5S1, trunk inclination angle, and trunk angular velocity. Results are shown for each assistance mode *no-exo* (green), *inclination* (red), *hybrid* (yellow), and *dynamic* (blue), execution speed (normal and fast) and object weight (7.5 and 15 kg). 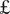 indicates the main effect of execution speed. # indicates the main effect of box weight. Bars with * indicate a significant posthoc differences between the assistance modes (*p*-value 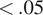 ).

Significant results are in bold (*p*-value <.05).

a Variables with significant main effect of the assistance. For these variables, a Bonferroni posthoc test was conducted for each combination of speed and box weight separately, to compare the assistance modes. Peak average and standard deviations are displayed in Figure 5.

For variables with a significant main effect of the assistance mode (*p*-value 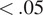 ), peaks average and standard deviation across all subjects are shown in Figure 5, for each assistance mode (*no-exo*, *inclination*, *hybrid*, *dynamic*), execution speed (normal and fast), and object weight (7.5 and 15 kg). For each combination of speed and box weight separately, Bonferroni posthoc tests were conducted to compare the effects of the assistance modes (significant differences between assistance modes are indicated by horizontal bars with *).

The total L5S1 moment was comparable for *hybrid* and *dynamic* strategies and *no-exo* modes, while it was slightly lower for the *inclination* strategy (on average less than 10 Nm), meaning that subjects slightly changed their lifting behavior. The net L5S1 moment generated by subjects was significantly lower with all the control strategies, for all object weights and execution speeds, with reductions ranging from 10 to 17%.

In line with this, reductions in peak back muscle activity with the exoskeleton were observed, ranging between 26 and 39% (i.e., percentage reduction compared with the *no-exo* mode) for the LL and the IL.

Slight reductions in trunk inclination were observed (ranging from 3 to 11%), with no statistical significance, for all the conditions with the exoskeleton. In line with this, the trunk velocity also decreased when using the exoskeleton, although on average less for the *dynamic* strategy (13%) than for the *inclination* and *hybrid* strategies (17%).

Figure 5 suggests that when wearing the exoskeleton, the L5S1 disc compression forces are reduced, ranging from 10 to 16% over control strategies. Although a main effect of the assistance mode was found using three-way ANOVA tests, statistical significance between the different assistance modes was not achieved with posthoc tests.

## Discussion

The net L5S1 moment generated by subjects was significantly lower when wearing the exoskeleton for all the assistance modes, with reductions of up to 17%, in line with Koopman et al. (2019c). The peak reductions observed were similar to those obtained when the PLAD passive device was tested (Frost et al., 2009), and greater than those with Laevo, a commercially available passive exoskeleton for which a similar study was conducted (Koopman et al., 2019a). Although the reduction in the net L5S1 moment is expected to be mainly due to the effect of the exoskeleton assistance, part of this reduction may be due to the changes in lifting behavior (i.e., reduced speed and inclination), which are suggested by the slight decrease in the total L5S1 moment.

The reduction of the peak compression force on the L5S1 disc is in line with the previous study (16%) (Koopman et al., 2019c) with no significant difference between the control strategies. Peak reductions were greater than the reductions obtained when testing the Laevo (8–9%) (Koopman et al., 2019a). With a 16% reduction of peak compressive force, the risk of back-related MSDs may be substantially reduced (Brinckmann et al., 1989); Waters et al., 1993).

Reductions in peak spinal muscle activity ranging between 26 and 39% for LL and IL are in line with the results obtained when testing the previous prototype (Huysamen et al., 2018; Toxiri et al., 2018) and other active devices (Muramatsu et al., 2013; Chen et al., 2018; Ko et al., 2018) and greater than the reductions obtained when testing the Laevo (Koopman et al., 2019a). Additionally, the decrease in muscle activity when using the exoskeleton might successfully reduce muscle fatigue (Potvin and Norman, 1993). An increase in the endurance time when using the exoskeleton is therefore expected. However, further investigations might detect whether the reduction in spinal muscle activation coincides with a shift from active to passive force generation (i.e., the extensor moment generated by passive tissues increases). In fact, although the contribution due to spinal muscles on lumbar compression is dominant, the passive contribution to the extensor moment also generates compression on the spine. Moreover, passive tissues could creep or incur microdamage (Solomonow et al., 2003).

Compared to our previous study on an earlier prototype, the reduction in peak lifting speed when using the exoskeleton was lower overall (Koopman et al., 2019c). This improvement may be partially attributed to the technical advancements of the exoskeleton (the weight of the new prototype is lower and the actuators have lower inertia). Moreover, the speed reduction for the *dynamic* control strategy appears to be lower compared to the other two strategies, although no statistical significance was found. This result encourages further investigation as it seems to support our initial hypothesis that the new control strategy provides more appropriate support to the tasks, improving the timing of the assistance in relation to the typical dynamics of the movement, with positive improvement in intuitiveness and comfort in use, and limiting the exoskeleton’s negative impact on productivity (i.e., the hindrance to fast movement is reduced). Lower speed reduction associated with the use of XoTrunk could be an advantage with respect to the use of passive exoskeletons such as the Laevo, for which a significant reduction of trunk angular velocity was observed (Koopman et al., 2019a).

Reductions in peak inclination angle between modes with the exoskeleton and the *no-exo* mode were around 11%. This slight reduction when wearing the exoskeleton is most likely because the structure of the device does somewhat hamper the lumbar flexion, resulting in a slight limitation of the freedom of movement. With respect to the state of the art, a greater reduction of user bending was seen in Näf et al. (2018), using a passive exoskeleton. On the other hand, other tests with the Laevo passive exoskeleton showed an increase in the flexion angle when using the exoskeleton (Bosch et al., 2016; Koopman et al., 2019a), but no results were reported for the trunk inclination angle. The slight reduction of the peak trunk inclination angles, however, may be interpreted as the exoskeleton still partially hindering subjects movement or influencing the lifting behavior. Thereby, part of the reduction of peak total L5S1 moment can be attributed to the reduction of the inclination. However, the fact that the exoskeleton affects the lifting behavior in a way that reduces the trunk inclination without preventing the execution of the movement may be seen as an advantage since the total L5S1 moment decreases, and thus the related risk of reporting a back injury decreases as well.

The improvement obtained in the present study may be partially attributed to the technical advancements of the exoskeleton. The weight of the new prototype as well as the actuators’ perceived inertia are lower. A further improvement of the new prototype is visible in Figure 4, that is, improved low-level control allows the actuators to track the reference torque more accurately, resulting in more effective assistance compared to the previously tested version (Koopman et al., 2019c). Despite the improvement, imperfect tracking of the reference torque by the exoskeleton actuators still affects the applied assistance. In particular, the applied torque at the beginning of lifting, when the speed increases rapidly, is substantially less than expected, correspondingly impacting the effectiveness. This is particularly inconvenient for the dynamic strategy, which critically increases the assistance when significant acceleration occurs.

One limitation of the present study is that only peak values were considered and not integral values. In particular, the difference between integral and peak values may be significant when comparing the normal and fast speeds. In fact, while peak compressive force and L5S1 moment are higher at fast speed, the cumulative values are much greater at the slow lifting speed because of the longer lifting duration (Greenland et al., 2013). Moreover, focus on peaks may have hidden some advantages of the *dynamic* control strategy. Figure 4 suggests meaningful differences especially during the upright phase.

Another limitation of the present study is that no predefined pace for the normal and the fast speed was set for executing the task. Therefore, the effects of the different strategies when changing the speed cannot be precisely analyzed (i.e., task speed is subjective and changes between subjects). In particular, we expect more hindrance during the lowering phase at fast speed with the *inclination* and the *hybrid* strategies. Indeed, at the beginning of lowering, the user must move against the assistance provided by the exoskeleton, that is, higher with the *inclination* and *hybrid* strategies than for the *dynamic.*

Considering its hardware implementation, the main drawback of the *dynamic* strategy is that an extra IMU has to be added on the user’s trunk. A further limitation regards the angular acceleration signal, that is obtained by differentiating and filtering the angular velocity. In particular, the design of the filter requires a trade-off between signal delay and noise. The filter frequency of 1 Hz has been selected to reduce the noise in the acceleration signal introduced by the differentiating of the angular velocity signal. However, the delay introduced in the assistance by signal filtering may be critical and has to be compared with the frequency of the movements performed by users. Moreover, overestimating the acceleration can lead to feedback inversion and instability (Calanca et al., 2017). To avoid excessive torques, the magnitude of the acceleration gain 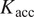 should be considered carefully. In this study, all the gains 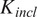 , 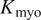 , and 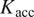 were kept fixed for all the subjects to allow comparison between subjects and to reduce the complexity. However, personalized and thus more effective assistance may be obtained with subject-specific gains. Future works will address gain tuning in order to adjust the assistance to subjects’ individual preferences (e.g., comfort and perceived pressure), body characteristics, and task conditions. In particular, subject-specific values may be set based on subject’s mass and standard anthropometrics. However, we believe that the user should retain the possibility to adjust the gains to a degree, to address his preferences in terms of perceived assistance and hindrance, and overall comfort.

Compared to passive devices, active exoskeletons have been associated with greater reductions in some of the factors that increase the risk of developing MSDs, as discussed above. A number of studies on active devices have shown a substantial reduction in the back muscle activity (Muramatsu et al., 2013; Chen et al., 2018; Huysamen et al., 2018; Ko et al., 2018; Toxiri et al., 2018), even greater than that obtained when the Laevo was tested with a similar procedure (Koopman et al., 2019a).

From an energetic point of view, active exoskeletons have the potential to add and deliver energy to the user. While the *inclination* controller is essentially passive, the *hybrid* and *dynamic* controllers might have a net energy injection over one cycle. These strategies thus would extend well to possible future exoskeletons capable of greater assistive torques, while existing strategies based on inclination or passive assistance may not apply equally well. In fact, for active devices that do not consider dynamic factors and for passive devices, an increase in the total torque supplied would result in increased hindrance while bending over during the lowering phase.

Future works will address the assessment of the users’ experience to compare the perceived comfort and intuitiveness between control strategies. Moreover, considering the use in industrial settings, improvements of the perceived fatigue and endurance time when using the exoskeleton could be valuable additional benefits that have to be investigated. Combinations of the control strategies presented will also be considered. In particular, the *dynamic* strategy, that acts so as to compensate for the inertia, could be improved by adding the information of the external load (e.g., measured with the Myo armband or a forcemyography sensor, as reported in Islam and Bai, 2019).

Additionally, the effects of the exoskeleton use on other parts of the body or side effects have to be further investigated. In fact, while back muscle activity was shown to be reduced using the exoskeleton, other muscles (e.g., leg or abdominal muscles) could increase their activity. As an example, in fully flexed postures, the activity of the abdominal muscles increases, as found for the Laevo during static holding tasks (Koopman et al., 2019b). However, in this work, we decided not to investigate the effects of our exoskeleton on the activation of leg and abdominal muscles. In fact, when our previous prototype was tested during lifting and lowering tasks, no significant difference was found in the abdominal muscle activation, while the biceps femoris activity was even reduced (Huysamen et al., 2018). Furthermore, also varying the lifting technique when using the exoskeleton may lead to significant changes in the activation of the different muscle groups (Frost et al., 2009). For this reason, and also to underline additional effects of the exoskeleton, changes in the lifting behavior should be better analyzed. For example, the fact that the lifting technique adopted when using the exoskeleton may reduce some risk factors (e.g., the lumbar flexion angle or the execution speed) without preventing the execution of the movement can be an additional advantage. While the focus of this work was on symmetric lifting and lowering tasks, the effects of the exoskeleton on reducing the asymmetry of the movement may be explored as well, as trunk twisting has been proven to increase the risk of developing low back pain (McGill 1991; Shan et al., 2013).

## Conclusions

Using the exoskeleton during lifting tasks reduced the compression force on the L5S1 disc, and correspondingly, the risk of developing back-related MSDs might decrease. Statistically significant reductions in peak lumbar extensor moment and peak spinal muscle activity were achieved when using the exoskeleton controlled with the three different strategies. Contrary to our expectations, substantial differences between the control strategies in the reductions of compression force, lumbar moment, and back muscle activation were not observed. Nonetheless, based on the results obtained, positive effects are expected with all the strategies in reducing the muscle effort and the overall exertion perceived by the user, as well as on increasing the endurance time.

The new control strategy reduced the movement speed less with respect to the other two strategies. Specifically, it improved the timing of the assistance in relation to the typical dynamics of the target task, and it reduced the hindrance to fast movement, thereby promoting intuitiveness and comfort.

## Data Availability

The data that support the findings of this study are available from the corresponding author, M.L., upon reasonable request.

## Supplementary material

For supplementary material accompanying this paper visit https://doi.org/10.1017/wtc.2020.8. click here to view supplementary material
